# Fast adaptive optics scanning light ophthalmoscope retinal montaging

**DOI:** 10.1364/BOE.9.004317

**Published:** 2018-08-15

**Authors:** Benjamin Davidson, Angelos Kalitzeos, Joseph Carroll, Alfredo Dubra, Sebastien Ourselin, Michel Michaelides, Christos Bergeles

**Affiliations:** 1 Wellcome/EPSRC Centre for Interventional and Surgical Sciences, University College London, London, UK; 2 Translational Imaging Group, Centre for Medical Image Computing, University College London, London, UK; 3 NIHR Biomedical Research Centre at Moorfields Eye Hospital and Institute of Ophthalmology, University College London, London, UK; 4 Medical College of Wisconsin, WI, USA; 5 Stanford University, CA, USA

## Abstract

The field of view of high-resolution ophthalmoscopes that require the use of adaptive optics (AO) wavefront correction is limited by the isoplanatic patch of the eye, which varies across individual eyes and with the portion of the pupil used for illumination and/or imaging. Therefore all current AO ophthalmoscopes have small fields of view comparable to, or smaller than, the isoplanatic patch, and the resulting images have to be stitched off-line to create larger montages. These montages are currently assembled either manually, by expert human graders, or automatically, often requiring several hours per montage. This arguably limits the applicability of AO ophthalmoscopy to studies with small cohorts and moreover, prevents the ability to review a real-time captured montage of all locations during image acquisition to further direct targeted imaging. In this work, we propose stitching the images with our novel algorithm, which uses oriented fast rotated brief (ORB) descriptors, local sensitivity hashing, and by searching for a ‘good enough’ transformation, rather than the best possible, to achieve processing times of 1–2 minutes per montage of 250 images. Moreover, the proposed method produces montages which are as accurate as previous methods, when considering the image similarity metrics: normalised mutual information (NMI), and normalised cross correlation (NCC).

## 1. Introduction

AOSLO is an optical imaging technique which combines Adaptive Optics (AO) and Scanning Light Ophthalmoscopy (SLO) to produce cellular resolution images of the retina *in vivo*, non-invasively [1]. AO is a technique by which wavefront aberrations are measured and then dynamically compensated for using a wavefront sensor, controller and actuated mirror. As such, AO can be applied to any ophthalmic imaging device which requires passing light into or out of the eye, but is typically used with SLOs as these produce the best contrast and lateral resolution [1]. Modern AOSLOs capture multiple images simultaneously from the same retinal location but using different modalities, such as confocal, non-confocal split-detection, and dark-field. In what follows we will refer to each of such image sets as a ‘tile’. In a typical imaging session 50–150 tiles are acquired. Metrics related to retinal substructures, such as cone photoreceptors, can be extracted from these images, and have been used to understand pathology and measure treatment efficacy [1,2].

**Fig. 1:**
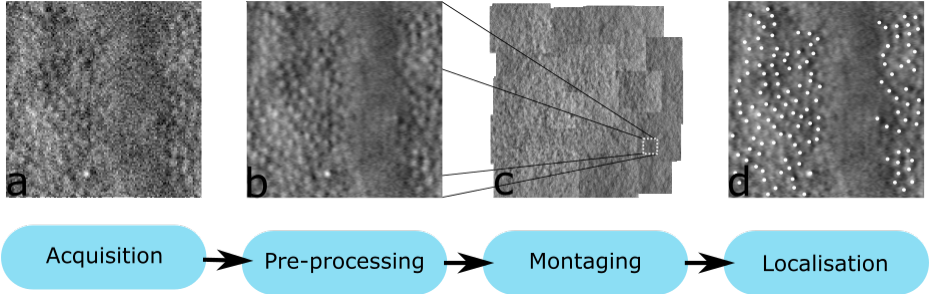
The standard image processing pipeline for cone photoreceptor AO retinal imaging. (a) Image acquisition typically produces low quality images that are (b) coregistered to improve the signal-to-noise ratio. (c) The registered images are then montaged to create a larger field of view of the retina. (d) Finally, the locations of cones are marked in images extracted from the montage.

Extracting this information from AOSLO images is time-consuming, costly and error-prone, due to the amount of manual image processing required. Multiple processing steps must be carried out, even before the lengthy manual montaging takes place (see Fig. 1). The large amount of manual work required to extract metrics from AOSLO imaging data is a significant limitation of the technology, as analysing large amounts of data, in longitudinal studies, or studies with large cohorts is impractical [2].

**Fig. 2:**
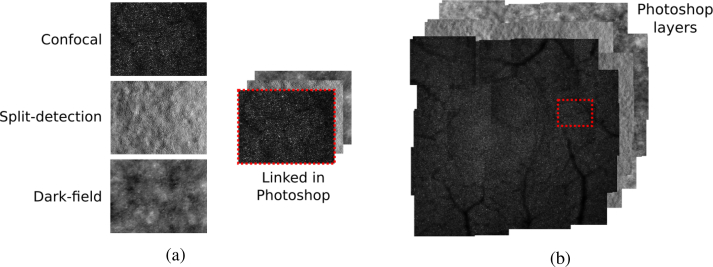
A retinal montage. (a) the three simultaneously acquired images from the same location. From top to bottom: confocal, split-detection, and dark field. These are collected together into a tile, in Photoshop these art layers are linked, so that moving one image, moves the other two; (b) completed retinal montage highlighting the position of the tile from (a). Each of the 3 montages, is a layer in Photoshop, allowing image analysts to easily move between the montages.

This paper focuses on the rapid automatic construction of retinal montages from AOSLO images, but the approach is applicable to other imaging modalities of the photoreceptor mosaic as well. This process is currently, often carried out manually in Photoshop (Adobe Creative Suite) or equivalent photo-editing software, whereby tiles are overlapped with each other like a jigsaw (see Fig. 2). Once complete, the montage is used to assess whether desired retinal coverage was achieved. If the montage is of sufficient quality, regions of interest (ROI) are extracted from the montage, and metrics such as cone density are extracted from each ROI. This allows to accurately determine the ROI’s retinal eccentricity relative to the fovea.

The state-of-the-art technique for automatic montaging, referred to as the SIFT approach hereafter, automatically constructs retinal montages but can take several hours to do so [3]. It relies on Scale Invariant Feature Transform (SIFT) feature-based image stitching to automatically construct AOSLO montages [4]. The strength of the approach is that it produces a highly accurate montage, even better than manual alignment in some cases. The long run time can be attributed to the use of SIFT keypoints and descriptors within the algorithm, which are known to have high computational overhead. We found, working closely with imaging specialists at Moorfields Eye Hospital, who are responsible for collecting and analysing AOSLO imaging data, those for whom the SIFT approach is designed, that the long processing required to construct a montage was preventing them from fully incorporating the technique into their workflow. Our approach is now used on a daily basis.

Henceforth, we address the long run time associated with the SIFT approach and present the fastest automatic montaging method to date, which can be inserted seamlessly into current workflows. Using Oriented Rotated fast Brief (ORB) features, which are faster to compute, together with an approximate nearest neighbours search and a modification of the underlying montaging approach [4], our method significantly reduces automatic montage-construction time, without sacrificing montage quality.

## 2. Materials and methods

The fast auto-montaging framework described in this work makes several key contributions to the SIFT approach. Our image stitching framework, also takes advantage of the three images in a tile and the availability of rough tile locations, collected during acquisition. First, we compute ORB features which are fast to calculate, as well as to compare; second, we use approximate nearest neighbour search, which further reduces the time it takes to compare whether two locations in an image appear similar. Finally we do not search for the best tile to align to another, we instead search for an alignment which is ‘good enough’, subject to a threshold. The effect of these alterations to the SIFT approach is detailed in Sec. 3, which demonstrates a significantly faster auto-montaging approach without compromise in quality.

This study was conducted in accordance with the tenets of the Declaration of Helsinki (1983 Revision) and the applicable regulatory requirements. After approval of the study and its procedures by the ethics committees of Moorfields Eye Hospital and University College London, informed consent was obtained from all participating subjects prior to enrolment.

### 2.1. AOSLO image data

A custom built AOSLO based on Scoles *et al.* [5] was used to acquire cellular resolution images of the photoreceptor mosaic. The imaging system produces sets of 3 image sequences captured simultaneously using the same light, and thus in perfect spatial registration, and from the same location, known as direct, reflect and confocal. These are combined, and, through a process of registration and averaging [5–7], produce the 3 images that form the tiles used here.

To ensure our algorithm is robust and accurate, data from subjects with different conditions were used. Specifically, we consider 9 datasets: 3 with no pathology, 2 from Stargardt disease (STGD) patients, 2 from subjects with achromatopsia (ACHM), and 2 with retinitis pigmentosa (RPGR mutation). The data sets included some that were of the lowest quality that our expert imaging specialists could manually montage. Finally, the data consisted of tiles of varying fields of view, either 1° × 1°, or 1.5° × 1.5° (see Table 1). This variation allowed us to validate that the proposed method produced high quality montages under typical and challenging scenarios.

**Table 1:** Description of data used to validate the proposed method.

Subject	Age	Condition	F.O.V (°)	Quality	# Tiles
MM_0105	24	Control	1	Good	81
MM_0020	10	STGD	1.5	Fair	85
MM_0303	12	Control	1	Fair	14
MM_0362	15	ACHM	1.5	Good	54
MM_0384	8	RPGR	1	Fair	46
MM_0389	14	RPGR	1.5	Good	67
MM_0410	25	Control	1, 1.5	Good	153
MM_0432	17	STGD	1.5	Good	98
MM_0436	10	ACHM	1	Fair	18

### 2.2. Feature-based image stitching

**Fig. 3:**
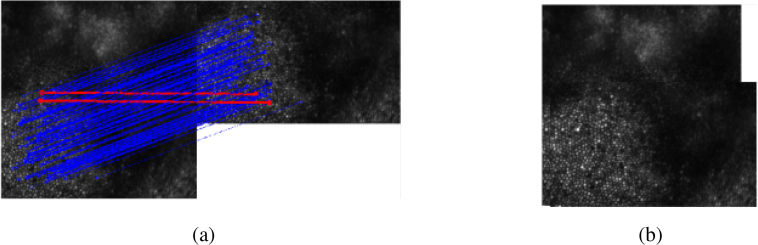
Stitching images using features. (a) Keypoints in each image (circles) and their corresponding matches (connected via a line). Note here there are two incorrect matches in red, which will be excluded after applying random sample consensus (RANSAC). (b) Result of aligning images using the calculated transformation.

The SIFT and the proposed method both montage tiles via feature-based image stitching. This montaging framework aligns images by calculating keypoints and descriptors in each image, which can then be used to estimate a transformation that aligns two given images (see Fig. 3). Keypoints can be thought of as locations of salience within an image, for example, corners, where there are sharp changes in image intensity, and descriptors as encoding the appearance of areas around keypoints. There is an associated descriptor to every keypoint, which allows to compare mathematically keypoint locations in two different images and determine their similarity. For example, let *k*_1_, *k*_2_ ∈ ℝ^2^ be keypoints in two separate images, and *d*_1_, *d*_2_ ∈ ℝ*^n^* the associated descriptors. Then if

(1) $$\text{distance}(d_{1},d_{2})<\lambda ,\;\;\;\;\;\;\lambda >0$$

we consider *k*_1_ and *k*_2_ to be the same location in two images. Using these pairs, a transformation, that aligns one image to the other can be estimated. To match descriptors we used the ratio test, which improves the accuracy of matches in comparison to using (1) [4]. This is achieved by considering the two closest matches to a given query descriptor. If the best match of the two is significantly better than the second-best, only then are the query and best match paired together.

**Fig. 4:**
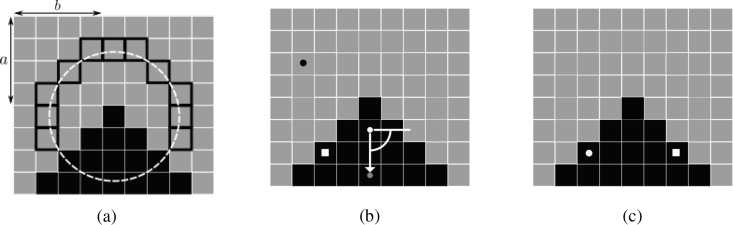
Computing ORB Features. (a) There are 11 contiguous pixels on the circular arc (white dashed) around pixel (*a*, *b*) which are lighter than it (pixels with bold edges). (b) An intensity centroid (grey circle) is calculated giving the keypoint an orientation; and an example pixel pair used to calculate the BRIEF descriptor (c) The location of pixel pairs after aligning to the orientation. Note the changing colours of the pixel pairs is only to ensure visibility of the points within the figure.

To locate keypoints using ORB, a modified Features from Accelerated Segment Test (FAST) corner detection step is carried out [8]. In FAST corner detection, a pixel at row *a* and column *b* is said to be a corner if there exists at least *η* = 9 contiguous pixels in a circular arc, of radius *r* = 3, around (*a*, *b*) such that for an intensity threshold *λ* = 21:

(2) $$I(a_{i},b_{i})>I(a,b)+\lambda ,\;\;\forall i\in \{1,2,\ldots ,\eta \},$$

or

(3) $$I(a_{i},b_{i})<I(a,b)-\lambda ,\;\;\forall i\in \{1,2,\ldots ,\eta \},$$

where *I*(*x*, *y*) denotes the brightness of a pixel at (*x*, *y*) and where (*a_i_*, *b_i_*) are all the pixels on the circular arc (see Fig 4). Detecting these corners quickly is achieved by only considering a subset of pixels on the circular arc. To determine which subset to consider a decision tree was learned, in the original work, by training on images drawn from the PASCAL dataset, for details see [8].

ORB modifies the FAST keypoint detection by computing keypoints at multiple scales, ranking the returned FAST corners according to a corner-ness score, retaining only the top *N* = 5000 keypoints, and attaching an orientation to each keypoint. This is to produce multi-scale keypoints and to eliminate edges, which can be returned from the standard FAST step. To compute keypoints at multiple scales a Gaussian image pyramid is used to downsample the image. At each scale of the pyramid, FAST corners are detected. To rank them, points are sorted according to a Harris corner measure, and the top *N* retained are the ORB keypoints [9]. Keypoints are then made rotationally invariant by attaching an orientation to each of them. This is calculated using an intensity centroid [10], so that the orientation is given by the direction from keypoint to centroid.

For each detected keypoint we then calculate ORB descriptors. ORB descriptors consist of a modified Binary Robust Independent Elementary Feature (BRIEF) descriptor [11]. A BRIEF descriptor for keypoint (*a*, *b*) consists of an *N_d_* = 256 binary vector, where the *i*^th^ element of the vector is determined by a binary test *τ_i_*:

(4) $$\tau_{i}(\left(a_{1}^{i},b_{1}^{i}\right),\left(a_{2}^{i},b_{2}^{i}\right)=\{\begin{array}{ll} 1 & I\left(a_{1}^{i},b_{1}^{i}\right)<I\left(a_{2}^{i},b_{2}^{i}\right)\\ 0 & I\left(a_{1}^{i},b_{1}^{i}\right)\geq I\left(a_{2}^{i},b_{2}^{i}\right) \end{array},$$

where ($a_{1}^{i}$, $b_{1}^{i}$) and ($a_{2}^{i}$, $b_{2}^{i}$) are a chosen pixel pair, from a patch of size 2*k* + 1 × 2*k* + 1 = 31 × 31 centred at (*a*, *b*). A machine learning step was used in [12] to find 256 pixel pairs which would give binary tests that were: uncorrelated and had high variance. This was to ensure that each test was informative, and discriminative respectively. For details, the reader is directed to [12]. ORB modifies this descriptor making it rotationally invariant, by aligning the pixel pairs to the previously computed orientations from the keypoint detection stage (see Fig. 4).

#### 2.2.1. Approximate nearest neighbour comparison

Another feature of our method is the use of approximate nearest neighbour search to further reduce algorithm run time. Local Sensitivity Hashing (LSH) was used, which rather than exhaustively compare a query vector to every possible neighbour, returns a highly likely neighbour [13]. To query a vector *q* against a set of vectors *V* in this manner we perform the following steps. Each member of *V* is placed into *b* = 6 buckets via functions *g_i_*. For example, *v*_0_ ∈ *V* could be placed in the buckets *g*_1_(*v*_0_), *g*_2_(*v*_0_) and *g*_3_(*v*_0_), if we choose to have each *v* ∈ *V* in 3 buckets. To query *q* against *V* we exhaustively compare *q* against *V̂*, such that

(5) $$\hat{V}=\left\{v\in \underset{i}{\cup }g_{i}(q)\right\}.$$

To ensure exact nearest neighbours are found with high probability, *g_i_* is chosen so that: if *q* is close to *v* then *g_i_*(*v*) = *g_i_*(*q*), and if *q* is not close then *g_i_*(*v*) ≠ *g_i_*(*q*). In this way only a subset of vectors in *V* are compared with *q*, reducing the time spent searching for nearest neighbours. Note that, if the nearest neighbour of *q* in *V* is not in ∪*_i_* *g_i_*(*q*), then it will not be found. In this instance only the nearest neighbour of *q* in the set ∪*_i_* *g_i_*(*q*) will be found, and returned as the approximate nearest neighbour.

### 2.3. Exploiting AOSLO image structure for montaging

There are two aspects of AOSLO imaging which are exploited in the SIFT approach. Accuracy is improved, by using the 3 image types in a tile to register it. Three sets of matching pairs are calculated for a given pair of tiles, one for each image type, and the union of these pairs is then taken. Therefore, when estimating a transformation many more matches can be used, even if features appear only in one of the image types. Random sample consensus (RANSAC) was used to estimate the aligning transformation between tiles, as well as assess the quality of the transformation [14]. In this step, *i* = 1000 samples of keypoint matches are used to estimate multiple registration transformations, which are then rated according to how well they map one set of keypoints from one tile to those in the other tile. The number of keypoints which match to within *δ* = 10 pixels of their corresponding match, known as the number of inliers, is used to assess if two tiles overlap at all. To improve speed, we can use information collected during image acquisition to reduce the number of required tile comparisons. For example, to naively montage a tile when there are *n* tiles in total, one would require (*n* − 1) comparisons, *i.e.* to compare the tile to every other to find the best alignment. However, during acquisition imaging specialists record the approximate location of the retina they are attempting to image, assuming consistently reliable fixation. Using this information, we are able to only check within tiles which are no more than a distance *d* = 7 from the location the tile itself was acquired. By searching for images which are within the distance *d* = 7 the approach can handle potential poor fixation or mistakes in location recording during image acquisition. Note, we refer to tiles which are close to each other in this manner as ‘nominally close’.

### 2.4. ORB montaging framework

In this section we discuss how tiles are aligned, and deal with the issue of discontinuous montages. To align tiles, the montage is constructed iteratively. First, a single random tile is considered as the tile-of-reference (ToF), and other tiles are aligned to it. If tiles are found for which quality transformations to the ToF can be estimated, then they are aligned to the ToF inside the montage. Another contribution of our method is immediately taking a ‘good’ transformation, rather than searching for the ‘best’. A good transformation is one for which there are *T*_1_ inlier matches. Such a transformation is taken immediately as the desired transformation, in contrast to the approach in [3], which always searches for the best. If no transformation is found with *T*_1_ matches, then if the best transformation has at least *T*_2_ inlier matches it is taken as the sought transformation. This process continues, attempting to align tiles to those currently in the montage, until either the montage is completed or it is not possible to estimate a quality transformation for an unaligned tile. In the case of being unable to align any remaining unaligned tiles, a random, unaligned tile is set as a new ToF, inside a different montage. This means there would be at least two distinct montages for this dataset. This is often the case in montage construction as there may not be a tile to connect to areas of the retina which have been successfully imaged. The whole process is repeated for the new montage. The algorithm continues until there are no more unaligned tiles. The proposed approach is given in detail in Algorithm 1.

**Algorithm 1:** Auto-Montaging with ORB

Input: Tiles *T* = {*t*_1_, *t*_2_, . . ., *t_n_*} with *t_i_* = (*c_i_*, *s_i_*, *d_i_*) consisting of three images	
Nominal positions *P* = {*p*_1_, *p*_2_, . . ., *p_n_*}	
Output: Transformations between images	
Calculate ORB keypoints and descriptors of all tiles	
Associate nominally close tiles	
**while** Still an unaligned image **do**	
Pick random unaligned tile and consider aligned	
**while** aligned a tile on previous iteration **do**	
**for** src ∈ unmatchedImages **do**	
**for** dst ∈ nominallyClose(src) ∩ matchedImages **do**	
Compare src and dst descriptors using LSH	
Estimate transformation, and inliers using RANSAC, saving result	
**if** #*inliers* > 50 **then**	▹ Translation is good enough
Save transformation from src to dst	
Exit for loop	
**if** best #*inliers* > 10 **then**	▹ Best translation is good enough
Save transformation from src to its best match	

### 2.5. Parameter values and implementation

The method described herein was implemented in Python and used the OpenCV library for ORB features computation and LSH comparisons. The parameters for ORB feature computation and comparison were the default parameters in OpenCV version 3.4 [15], except that we required 5000 keypoints to be found. The remaining parameters were found through experimentation on the MM_0105 dataset, were fixed for all experiments, and are given in Table 2. The only parameter we found our approach to be sensitive to were the 5000 keypoints returned by ORB. Returning fewer keypoints seemed to increase the number of disjoint pieces created by our approach. The routine use of our software in the AO pipeline at Moorfields Eye Hospital further highlights its robustness to parameters.

**Table 2:** Parameters

Parameter	Value	Description

*i*	1000	RANSAC iterations
*δ*	10	RANSAC match threshold
*d*	7	Nominally close distance
*T*_1_	50	Immediately accept threshold
*T*_2_	10	Best match accept threshold
*N*	5000	Number of ORB features
*λ*	21	ORB brightness threshold
*η*	9	ORB contiguous pixels
*r*	3	Radius of circular arc
*b*	6	Number of LSH buckets

## 3. Results

In this section we show that the developed method improves average construction time by a factor of 16 when compared to the SIFT method, whilst having comparable scores when image similarity metrics were considered. We found that, of the data considered, our approach was a minimum of 5.5 times faster than the SIFT approach: ORB taking 158 s, whilst SIFT Took 876 s. Note that all experiments were run on a laptop computer with 8GB RAM and i7 processor.

### 3.1. Comparison to state-of-the-art

**Fig. 5:**
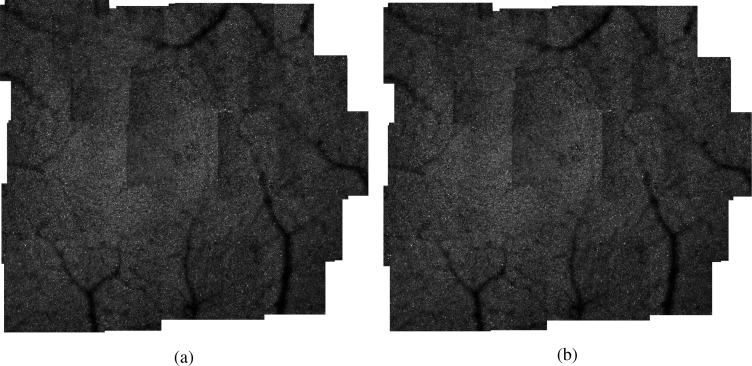
Montages constructed with both methods, (a) built using the proposed method, (b) built using the SIFT method.

We found an average construction time of 2 minutes for the proposed approach, whilst the average time for the SIFT method was 27 minutes. The difference in maximum construction times found were 5 minutes using the proposed approach and 93 minutes when using SIFT. To measure the accuracy of our approach we used the Normalised Cross Correlation (NCC), Normalised Mutual Information (NMI) and counted how many disjoint pieces each method returned. We found the accuracy of our method to be as good as the accuracy of the SIFT approach with both methods scoring similarly when the NCC and NMI was considered. Of note is that our method returns more disjoint pieces. This can be attributed to the increased overlap that is required to find a match (see Sec. 3.2). Practically, however, this does not add a significant amount of time to the construction of the montage, which has to be manually checked following the output of either algorithm (see Sec. 3.3). An example of a montage constructed with both methods is given in Fig. 5.

**Table 3:** Characterisation of algorithm performance. For each dataset this table shows the time each method took to construct the registrations, as well as how accurate each output montage was. Normalised cross correlation (NCC), normalised mutual information (NMI)

Montage	ORB	SIFT	ORB	SIFT	ORB	SIFT	ORB	SIFT
	Seconds	Seconds	NCC	NCC	NMI	NMI	# Pieces	# Pieces
MM_0105	75	3001	0.49	0.48	0.10	0.10	1	1
MM_0020	100	1970	0.60	0.62	0.13	0.14	9	4
MM_0303	3	107	0.82	0.87	0.21	0.24	1	1
MM_0362	158	876	0.56	0.60	0.12	0.13	6	4
MM_0384	69	806	0.55	0.61	0.12	0.13	3	1
MM_0389	92	1302	0.36	0.33	0.10	0.10	2	2
MM_0410	298	5577	0.42	0.40	0.10	0.10	1	1
MM_0432	95	841	0.54	0.54	0.10	0.11	6	3
MM_0436	36	309	0.34	0.39	0.10	0.09	6	6

### 3.2. Image overlap required to match

**Fig. 6:**
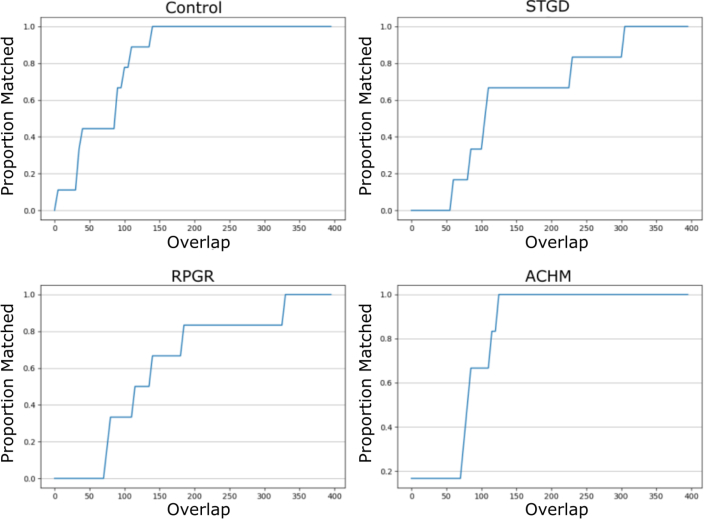
Proportion of tile pairs correctly matched with a given overlap.

As in Chen *et al.* [3], we investigated how much overlap was required to match two tiles. Tile pairs which overlapped by at least 350 columns or rows were identified in each dataset. To determine how much overlap was required to match a pair, a single tile from the pair was cropped to reduce the overlap, and then the tiles were matched. Two such pairs were taken from each dataset, and the proportion of correct matches, at each overlap, per-condition, were recorded. We found similar results to Chen *et al.* in that the control condition required the least amount of overlap, whilst pathological datasets required more. The proposed approach did, however, require a larger overlap than the SIFT approach. This explains the larger number of disjoint montage pieces returned by the proposed approach (see Table 4, Fig. 6).

**Table 4:** Size in pixels (p) of row or column overlaps required to ensure 100% matching of all tile pairs considered. Retinitis Pigmentosa (RPGR), Stargardt Disease (STGD), Achromatopsia (ACHM), Central Serious ChorioRetinopathy (CSCR). NA here indicates these conditions were not present in the data considered.

	Control (p)	RPGR (p)	STGD (p)	ACHM (p)	CSCR (p)
Proposed	140	305	330	125	NA
SIFT	75	250	NA	NA	100

The increased overlap required is due to the fact that ORB features cannot be computed as close to the edge of an image, unlike SIFT features. This is a result of how keypoints are computed in both methods. In SIFT, each pixel can be considered, whilst in ORB many edge pixels cannot be considered due to the 2*k* + 1 × 2*k* + 1 patch required to determine corner-ness. Because of this, only pixels which are at least 15 pixels away from each edge can be considered as keypoints. Moreover, ORB keypoints have been shown empirically to be found mostly in the central portion of an image [16].

### 3.3. Time to manually adjust

To investigate the effect that having more disjoint montage pieces has on total montage construction time, we measured how long it took to fine-tune and complete the montages by hand, following each algorithm’s output. A single expert human grader was given the Photoshop document output by each algorithm, for all data sets where there was a discrepancy in the number of pieces. These incomplete montages were then finalised by hand, checking for incorrect matches, and stitching together any tiles which were missed; and the elapsed time was measured. The ORB montage was always constructed first, so that any positive bias in timing, which could be gained by remembering a previously constructed montage, would only benefit the SIFT method. We found that, of the four datasets considered, ORB took longer to complete in 2 cases, SIFT in 1 case, and in one case the montages could not be completed by hand due to image quality (see Table 5). Also, of the tiles which were matched, no incorrect matches were found.

**Table 5:** Times, in seconds, to complete each montage by hand, when ORB produced a different number of disjoint pieces than SIFT. Not completed (NC)

Subject	ORB (s)	SIFT (s)

MM0020	NC	NC
MM0362	729	203
MM0384	246	30
MM0432	760	1000

### 3.4. Effect of ORB, LSH and finding ‘good enough’ alignments

In this section we show the speed-up granted by each of our contributions to the SIFT approach. The effect of each contribution is shown by montaging MM_0105, using various algorithmic setups. We found that ORB keypoints and descriptors were calculated on average, for a single tile, in 0.07 s; SIFT features took 4.08 s, on average to calculate. LSH cannot naively be used with SIFT descriptors, as the coordinates of the descriptors are not uniformly distributed [17]. Due to this we compared the speed of matching ORB features using standard, brute-force nearest neighbours, and LSH. Doing so we find that with LSH it takes 0.17 s to compare 2 tiles; whereas with a brute-force approach it takes 0.28 s. Finally, the effect of accepting a transformation with *T*_1_ = 50 inlier matches immediately was investigated. Montaging MM_0105 using ORB features, LSH and immediately accepting took 75 s; repeating this, whilst looking for the best transformation took 308 s.

## 4. Conclusions

Using fast to compute keypoints and descriptors, approximate nearest neighbour search, and by requiring only ‘good enough’ transformations, we were able to develop an accurate, auto-montaging method which is up to 16 times faster than the state-of-the-art. Despite the increased speed of the algorithm we maintained overall montage quality. Therefore, we have produced a highly applicable tool, which can reduce the image processing burden currently faced by those using AOSLO in their imaging studies. Our approach is also written in Python, and therefore does not require the purchase of any proprietary software. Code 1 is available at [18]. We have built an easy to use tool which fits seamlessly into current workflows. Eliminating a key limitation of previous methods, our work will help AOSLO imaging to reach its clinical potential.
